# Angiotensin II enhances the proliferation of Natural Killer/T-cell lymphoma cells via activating PI3K/Akt signaling pathway

**DOI:** 10.1042/BSR20202388

**Published:** 2020-10-13

**Authors:** Gui-Hua Zhang, Fa-An Miao, Jin-Ge Xu, Yan Zhang

**Affiliations:** 1 Department of Hematology, The Second Affiliated Hospital of Xuzhou Medical University, General Hospital of Xuzhou Coal Mining Group, Xuzhou, China; 2 Department of Neurosurgery, Affiliated Hospital of Xuzhou Medical University, Xuzhou, China; 3 Department of Hematology, The Affiliated Changzhou No.2 People's Hospital of Nanjing Medical University, Changzhou, China

**Keywords:** Angiotensin II, lymphoma, phosphatidylinositol 3-kinase, proliferation, protein kinase B

## Abstract

The present study was to determine the roles of Angiotensin (Ang) II in the growth of lymphoma in nude mice and the proliferation and viability of the human Natural Killer/T (NK/T)-cell lymphoma cell line SNK-6, and the activation of downstream signaling pathway. Lymphoma samples and corresponding normal tissues were obtained from lymphoma patients. Proliferation of SNK-6 cells was detected by CCK8 or MTT assay. The levels of Ang II and its receptor Ang II type 1 receptor (AT1R) were higher in lymphoma tissues than those in control tissues. Ang II increased the lymphoma volume and size in nude mice, the proliferation and viability and the proliferating cell nuclear antigen (PCNA) and Ki67 levels of SNK-6 cells. Losartan, an antagonist of AT1R, reduced lymphoma volume and size in nude mice, and the proliferation and viability and the PCNA and Ki67 levels of SNK-6 cells. The levels of phosphorylated phosphatidylinositol 3-kinase (p-PI3K) and phosphorylated protein kinase B (p-Akt) were increased by Ang II and then reduced by losartan in SNK-6 cells. The proliferation and viability of SNK-6 cells were increased by Ang II, but these increases were inhibited by PI3K inhibitor wortmannin and Akt inhibitor MK2206. The increases of PCNA and Ki67 induced by Ang II were inhibited by wortmannin or MK2206 in SNK-6 cells. These results indicate that Ang II/AT1R is activated in lymphoma, and Ang II promotes the progression of lymphoma in nude mice and the proliferation and viability of SNK-6 cells via activating PI3K/Akt signaling pathway.

## Introduction

Arising from extranodal lymphoid tissue or lymph glands, malignant lymphoma shows a growing morbidity globally [1]. Natural Killer T (NK/T)-cell lymphoma is Non-Hodgkin (the other subtype being Hodgkin’s lymphoma) and heterogeneous, with an aggressive clinical course [2]. NK/T-cell lymphoma varies in immunophenotype, location, morphology and genetics.

The renin–angiotensin system (RAS) affects tumor growth and migration by remodeling the tumor microenvironment [3]. RAS inhibitors, widely prescribed for cardiovascular diseases, have shown considerable anticancer potential [4,5]. Angiotensin (Ang)-(1–7) inhibits cell proliferation, migration and invasion by activating autophagy, which provides a possible treatment option for nasopharyngeal carcinoma (NPC) and recurrent NPC [6]. Expression of Ang II, a crucial biological peptide in the RAS, is closely associated with the development of cancer [7,8]. Ang II produced biological effects via its receptor Ang II type 1 receptor (AT1R) [9,10]. Telmisartan, a specific AT1R blocker, can effectively inhibit the growth of non-small cell lung cancer A549 cell line [11]. Activation of (pro)renin receptor ((P)RR) and AT1R is associated with the pathogenesis of conjunctival extranodal marginal zone B-cell lymphoma (EMZL) [12]. However, whether Ang II regulates lymphoma growth, and inhibition AT1R attenuates lymphoma remains unanswered.

Phosphoinositide 3-kinase (PI3K)/mechanistic target of rapamycin (mTOR) inhibitor BEZ235 showed an obvious anti-lung-cancer effect either as a support of chemotherapy or targeted therapy, or as a monotherapy [13]. Increased phosphorylation of protein kinase B (Akt) expression was involved in the overgrowth for esophageal cancer cell [14]. Phosphorylated AKT and MAPK were increased in copanlisib-resistant B-cell lymphoma cells [15]. PI3K inhibition with copanlisib or duvelisib continues to be an invaluable tool in the therapy of patients with lymphoid malignancies [16]. These findings inspired us to design the present study to explore whether Ang II promotes the proliferation of lymphoma relying on PI3K/Akt signaling pathway

## Materials and methods

### Clinical samples

Twelve pairs of NK/T-cell lymphoma samples (cervical lymph node) and normal adjacent samples (>10 cm away from the edge of the cancer) [17] were collected from the Hematology department of the Second Affiliated Hospital of Xuzhou Medical University from May 2017 to June 2018 (median age 63 years, range 51–76 years, six males and six females). Excluded were tissues that had been treated with chemotherapy or radiotherapy prior to surgery. The included tissue samples should demonstrate a tumor cellularity of at least 80% and clear viable tumor cells. The pathological stage and grade were appraised by an experienced pathologist. All the tissue samples were stored at −80°C until being used. All the patients had been clinically staged with endoscopic ultrasonography and multislice spiral computed tomography and had not received any chemotherapy or radiotherapy prior to surgery. The study was approved by ethics committee of the Second Affiliated Hospital of Xuzhou Medical University (approval number 20180321), and all subjects had signed the informed consent files.

### Animals

Experiments were carried out using 6-week-old male normotensive BALB/c nude mice (Vital River Biological Co., Ltd, Beijing, China) in the Animal Core Facility of Xuzhou Medical University. All the procedures were approved by the Experimental Animal Care and Use Committee of Xuzhou Medical University, and were conducted in accordance with the Guide for the Care and Use of Laboratory Animals (NIH publication No. 85-23, revised 1996). The mice were kept in a temperature-controlled room in a 12 h light–dark cycle with free access to standard chow and tap water. The related experiments were conducted at Experimental Animal Center of Xuzhou Medical University.

### Cell culture

The human NK/T-cell lymphoma cell lines SNK-6 (ATCC) were cultured in RPMI-1640 (BioChannel Biological Technology Co., Ltd.) supplemented with 10% fetal bovine serum (FBS; BioChannel Biological Technology Co., Ltd.) and 700 U/ml interleukin-2 (IL-2), and incubated at 37°C and 5% CO_2_. Losartan (an antagonist of AT1R [18]; 10^−5^ M), wortmannin (an inhibitor of PI3K [19]; 10^−7^ nM, Selleck, Shanghai, China) or MK2206 (an inhibitor of Akt [20]; 5 × 10^−6^ μM, Selleck) pretreatment lasted for 30 min, then, Ang II (10^−6^ M) was added.

### Nude mice xenograft experiments

SNK-6 cells were pre-treated with PBS (BioChannel Technology Co., Ltd., Nanjing, China), Ang II (100 nM, Sigma, MO, U.S.A.), losartan (2 μM, Sigma) or Ang II+Losartan in serum-free medium for 24 h prior to injection [21]. Therefore, the mice were randomly divided into four groups injected with SNK-6 cells treated with the above reagents. Tumors were implanted through the subcutaneous injection of SNK-6 cell suspension (about 10^7^) in PBS into the right flank of the mice. Then, Ang II or losartan intraperitoneally injected into the mice every day. Tumor size was measured every 5 days with electronic caliper. Tumor volume (*V*) was calculated by the formula: *V* = 0.5 × length × width^2^ [22]. After 30 d, the mice were cervical dislocation after anesthetized with isoflurane (2.5%). Death was confirmed by the absence of heartbeat, and corneal reflexes and paw withdrawal response to a noxious pinch. Tumor samples were collected and weighed in all groups.

### MTT assay

The viability of SNK-6 cells was detected using MTT assay (Sigma). The cells (10^4^ cells / well) were cultured onto 96-well plates with 100 μl of growth medium at 37°C with 5% CO_2_. After being culture for 24 h, the medium was replaced with fresh culture medium containing 0.5 mg/ml MTT dye. After 4 h of incubation at 37°C, the MTT solution was replaced with 150 μl of DMSO. The absorbance at 490 nm was then detected with a microplate reader (BioTek, VT, U.S.A.).

### Cell Counting Kit-8 (CCK-8) proliferation assay

SNK-6 cell suspension (100 µl) was seeded into 96-well plate (1000 cells/well). Four groups were set in this assay: PBS group, Ang II group, Losartan group and Losartan+Ang II group. Then, the cells were cultured in a CO_2_ incubator and the cell viability was detected at 24, 48, and 72 h. A total of 10 µl of CCK-8 (Solarbio Science & Technology, Co., Ltd., Beijing, China) reagent were added into each well and the plates were incubated at 37°C for 1.5 h. The optical density value was measured at 450 nm using a microplate reader (BioTek, VT, U.S.A.) and a proliferation curve was plotted.

### Cell counting

SNK-6 cells were seeded into 96-well plate (1000 cells/well). Prepare hemocytometer by cleaning surface and glass cover with 70% ethanol. After associated treatment, cells were counted using a automated cell counter (Countstar, Ruiyu Biotech Co., Ltd, Shanghai, China).

### Quantitative real time-PCR (qRT-PCR)

The total RNA in samples was extracted with Trizol (Ambion, TX, U.S.A.). In brief, total RNA was extracted using TRIzol® (Invitrogen; Thermo Fisher Scientific, Inc.). cDNA was synthetized from RNA via reverse transcription using random primers in a total volume of 10 μl, according to the instructions of the PrimeScript™ RT Master Mix (37°C, 15 min; 85°C, 5 s; Takara Biotechnology Co., Ltd.). The mRNA levels of PCNA and Ki67 were determined with SYBR Green I fluorescence. All cDNA was stored at −80°C before use. mRNA levels were determined via Power SYBR Green PCR Master Mix (Thermo Fisher Scientific, Inc.). All samples were amplified in triplicates for 40 cycles in a 384-well plate (95°C, 15 s; 60°C, 1 min) with a machine (Applied Biosystems, CA, U.S.A.). The relative gene expression was determined using the 2^−ΔΔCq^ method [23]. The relative gene expression was determined by calculating the values of Δcycle threshold (Δ*C*t) as a relative quantity to the endogenous control. The primers are shown in Table 1.

**Table 1:** List of utilized primers for qRT-PCR

Gene	Forward primer	Reverse primer
PCNA	CCTGCTGGGATATTAGCTCCA	CAGCGGTAGGTGTCGAAGC
Ki67	GCCTGCTCGACCCTACAGA	GCTTGTCAACTGCGGTTGC
GAPDH	CACCCACTCCTCCACCTTTG	CCACCACCCTGTTGCTGTAG

Abbreviations: GAPDH, glyceraldehyde-3-phosphate dehydrogenase; PCNA, proliferating cell nuclear antigen.

### Western blotting

NK/T-cell lymphoma samples and corresponding normal tissues were lysed in RIPA buffer. After electrophoresis and transfer to a nitrocellulose membrane, the proteins were probed with AT1R (1:2000, ab124734, Abcam, MA, U.S.A.), proliferating cell nuclear antigen (PCNA, 1:2000, ab19166, Abcam), Ki67(1:5000, ab92742, Abcam), p-PI3K (1:500, ab182651, Abcam), PI3K (1:1000, #4249, CST, MA, U.S.A.), p-Akt (1:2000, #4060, CST), Akt (1:1000, #4691, CST) primary antibodies, followed by incubation with secondary antibodies (Abcam, MA, U.S.A.). The bands were visualized using the enhanced chemiluminescence (ECL) substrate (BioChannel Biological Technology Co., Ltd.). The protein level was normalized to the protein level of glyceraldehyde 3-phosphate dehydrogenase (GAPDH; 1:10000, ab18602, Abcam).

### Ang II determination

NK/T-cell lymphoma samples and paired normal tissues were homogenized in lysis buffer and centrifuged. The total protein in the supernatant was extracted and measured using a BCA protein assay kit (BioChannel Biological Technology Co., Ltd.). The level of Ang II was measured using an enzyme-linked immunosorbent assay (ELISA) kit (USCN Life Science Inc., Wuhan, China).

### AT1R down-regulation

Recombinant Adenovirus-shRNA-AT1R (Ad-shRNA), and its negative control adenovirus (Ad-GFP) were both constructed and packaged by Genechem Co. (Shanghai, China). For knockdown experiments *in vitro*, 200 μl original solution (2 × 10^8^ transducing units/ml) was diluted in 2 ml Enhance Infection Solution (Genechem Co., Shanghai, China) to make the stock solution. When SNK-6 cells spread to 70–80% of the cultural dishes, the complete medium was removed. The cells were then transfected with serum-free medium containing 8 μl stock solution of Ad-shRNA/Ad-GFP per 1ml. After harvest for 8 hours, the medium was replaced with complete medium followed by washing with PBS for three times.

### Statistical analyses

Data were presented as the mean ± standard error of the mean (SEM) and analyzed using GraphPad Prism 7.0 (GraphPad software Inc., CA, U.S.A.). Statistical significance among two groups was evaluated by *t*-test and multiple groups was evaluated by one-way analysis of variance (ANOVA) with the Bonferroni post-hoc test. A two-tailed *P*-value <0.05 was considered statistically significant.

## Results

### Levels of Ang II and AT1R

**Figure 1:**
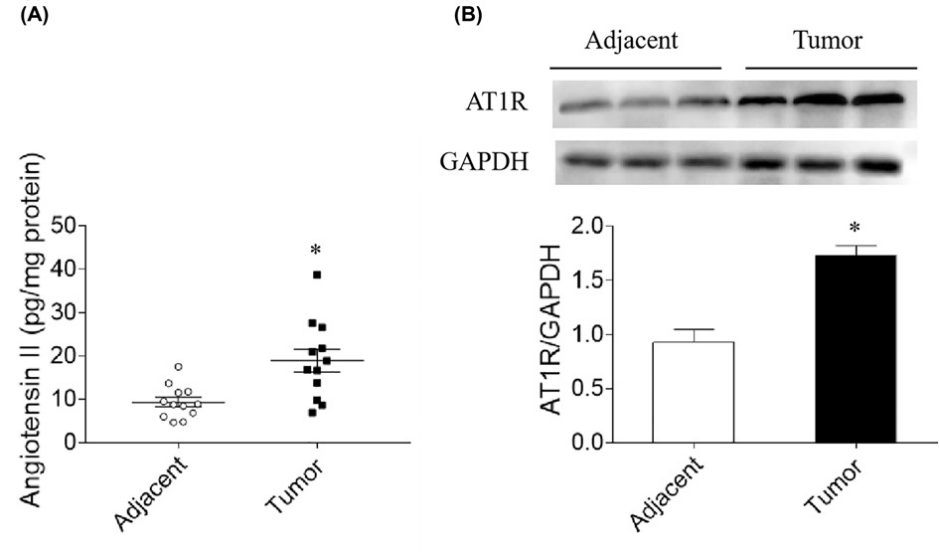
Levels of angiotensin (Ang) II and Ang II type 1 receptor (AT1R) in lymphoma tissue (**A**) Ang II level was higher in lymphoma tissue than in control tissue. (**B**) AT1R level was higher in lymphoma tissue than in control tissue. Results are expressed as mean ± SEM; *N*=12; *P<0.05 versus the adjacent group.

Ang II level was higher in lymphoma tissue than in control tissue (18.9 ± 2.6 vs. 9.4 ± 1.1 pg/mg protein, *P*=0.023) (Figure 1A). AT1R expression level in lymphoma tissue was 1.9 times of that in control tissue (*P*=0.019) (Figure 1B). Ang II can be detected in SNK-6 cells (36.6 ± 4.7 pg/mg protein).

### Effects of Ang II and losartan on lymphoma volume and weight

**Figure 2:**
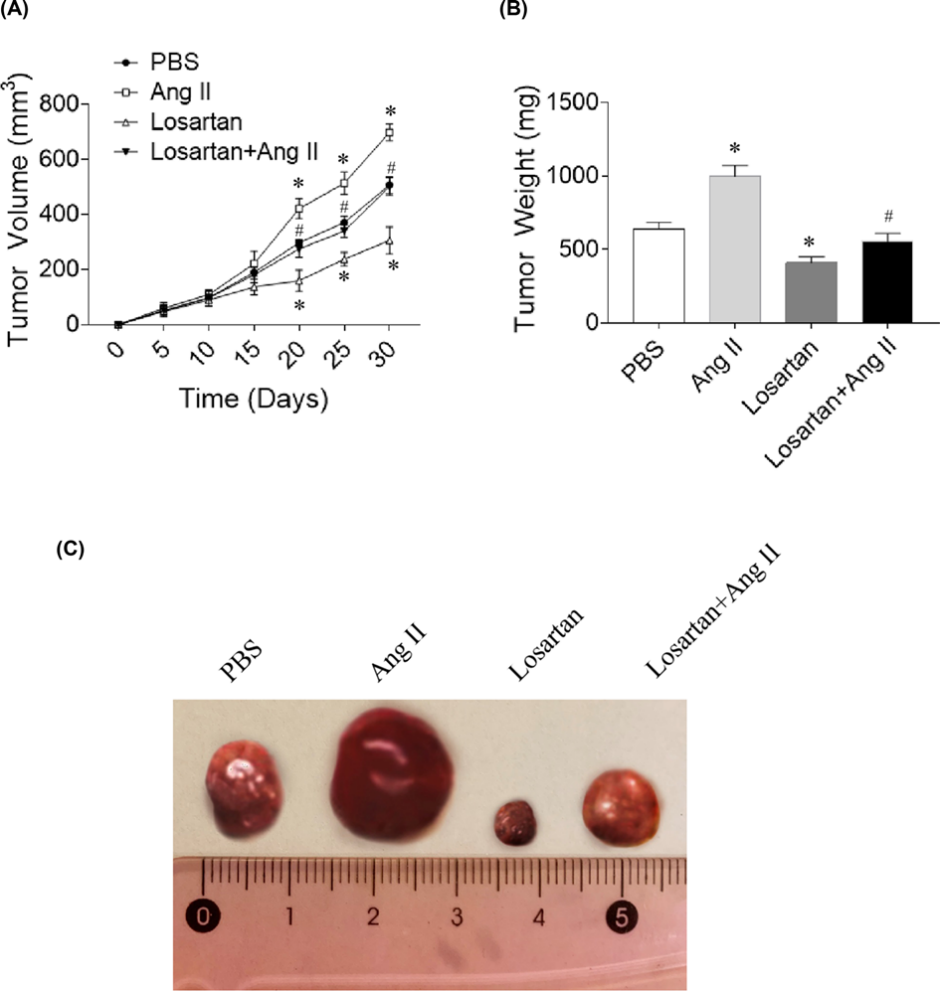
Effects of angiotensin (Ang) II and losartan on lymphoma volume and weight (**A**) Ang II increased, but losartan reduced lymphoma volume in nude mice. (**B**) Ang II increased, but losartan reduced lymphoma weight in nude mice. (**C**) The representative photograph showed the tumor tissues in four groups. The mice were treated with Ang II or losartan for 30 days. Results are expressed as mean ± SEM; *N*=8 for each group. *P<0.05 versus the PBS group; ^#^*P*<0.05 versus the Ang II group.

Ang II treatment increased the volume of tumor at Day 20 (*P*=0.035). The mean tumor weight increased after 30 d of treatment compared to that of PBS-treated control (*P*=0.024). After being treated with AT1R antagonist losartan for 20 d, the tumor volume was significantly reduced (*P*=0.031); After a treatment for 30 d, the mean tumor weight was significantly reduced (*P*=0.009). Losartan inhibited the elevation in tumor volume (*P*=0.041) and weight (*P*=0.023) induced by Ang II treatment (Figure 2A,B). The representative photographs of the tumor tissues in four groups are presented in Figure 2C.

### Effects of Ang II and losartan on SNK-6 cell proliferation and viability

**Figure 3:**
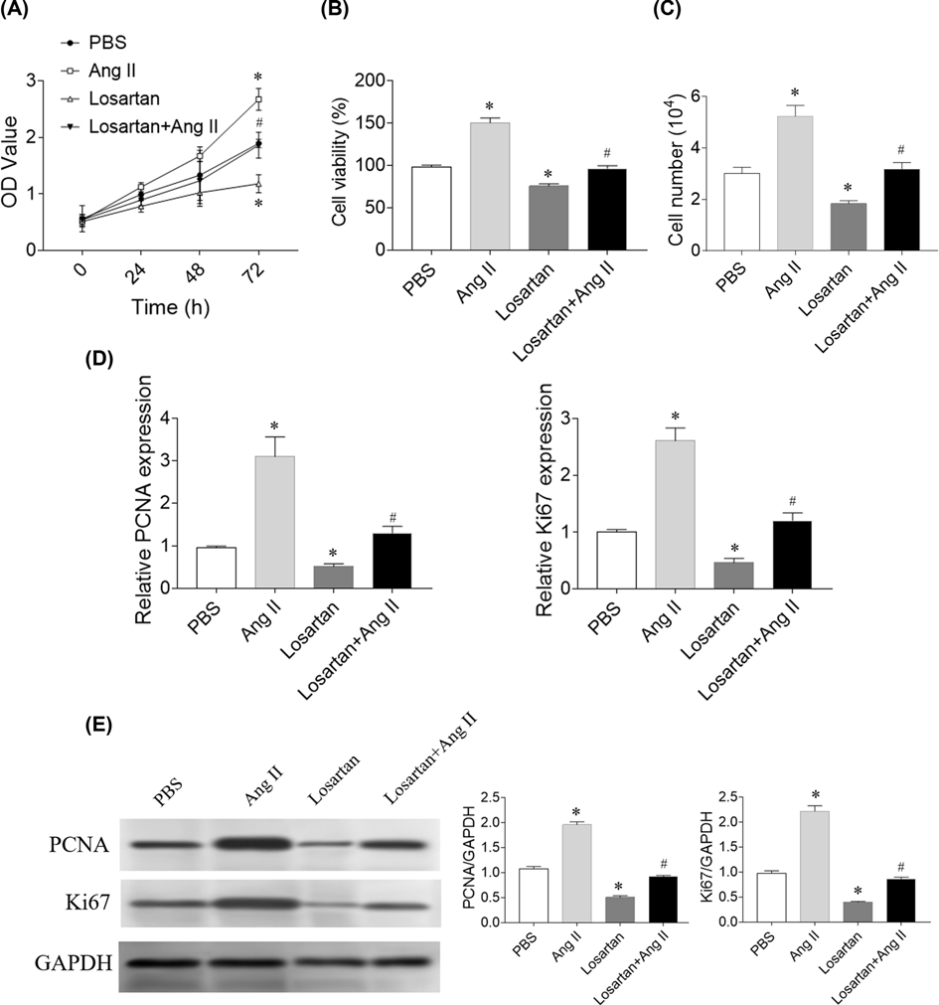
Effects of angiotensin (Ang) II and losartan on SNK-6 cell proliferation and viability (**A**) Ang II increased, but losartan attenuated SNK-6 cell proliferation according to the results of CCK-8 assay. (**B**) Ang II increased, but losartan attenuated SNK-6 cell viability according to the results of MTT assay. (**C**) Ang II increased, but losartan attenuated SNK-6 cell numbers. (**D**) Ang II increased, but losartan reduced proliferating cell nuclear antigen (PCNA) and Ki67 mRNA levels in SNK-6 cells. (**E**) Ang II increased, but losartan reduced proliferating cell nuclear antigen (PCNA) and Ki67 protein levels in SNK-6 cells. SNK-6 cells were treated with Ang II or losartan for 24 h. Three independent times experiments were repeated. Results are expressed as mean ± SEM. *P<0.05 versus the PBS group; ^#^*P*<0.05 versus the Ang II group.

Human NK/T-cell lymphoma cell line SNK-6 was treated with Ang II and losartan. The proliferative rate and viability of NK/T-cell lymphoma cells was detected using CCK-8 and MTT. CCK-8 results showed that Ang II significantly increased (*P*=0.012) and losartan significantly reduced (*P*=0.022) the proliferation of SNK-6 cells at 72 h. Losartan inhibited the proliferation of SNK-6 cells increased by Ang II treatment (*P*=0.033) (Figure 3A). MTT results showed that Ang II significantly increased (*P*=0.023) and losartan significantly reduced (*P*=0.018) the viability of SNK-6 cells at 72 h. Losartan inhibited Ang II-promoted the viability of SNK-6 cells (*P*=0.039) (Figure 3B). Cell number was increased by Ang II treatment (*P*=0.002), and reduced by losartan (*P*=0.001). Losartan significantly inhibited the increase of cell number induced by Ang II (*P*=0.010) (Figure 3C). PCNA (*P*=0.012) and Ki67 (*P*=0.009) mRNA levels were increased in SNK-6 cells treatment with Ang II, and were reduced in SNK-6 cells treatment with losartan (*P*=0.027 of PCNA and *P*=0.042 of Ki67). Losartan inhibited the increases of PCNA (*P*=0.011) and Ki67 (*P*=0.038) mRNA levels in SNK-6 cells induced by Ang II (Figure 3D). PCNA (*P*=0.027) and Ki67 (*P*=0.019) protein levels were increased in SNK-6 cells treatment with Ang II, and were reduced in SNK-6 cells treatment with losartan (*P*=0.041 of PCNA and *P*=0.039 of Ki67). Losartan inhibited the increases of PCNA (*P*=0.037) and Ki67 (*P*=0.024) protein levels in SNK-6 cells induced by Ang II (Figure 3E).

### Effects of ATIR down-regulation on the roles of Ang II in SNK-6 cell proliferation and viability

**Figure 4:**
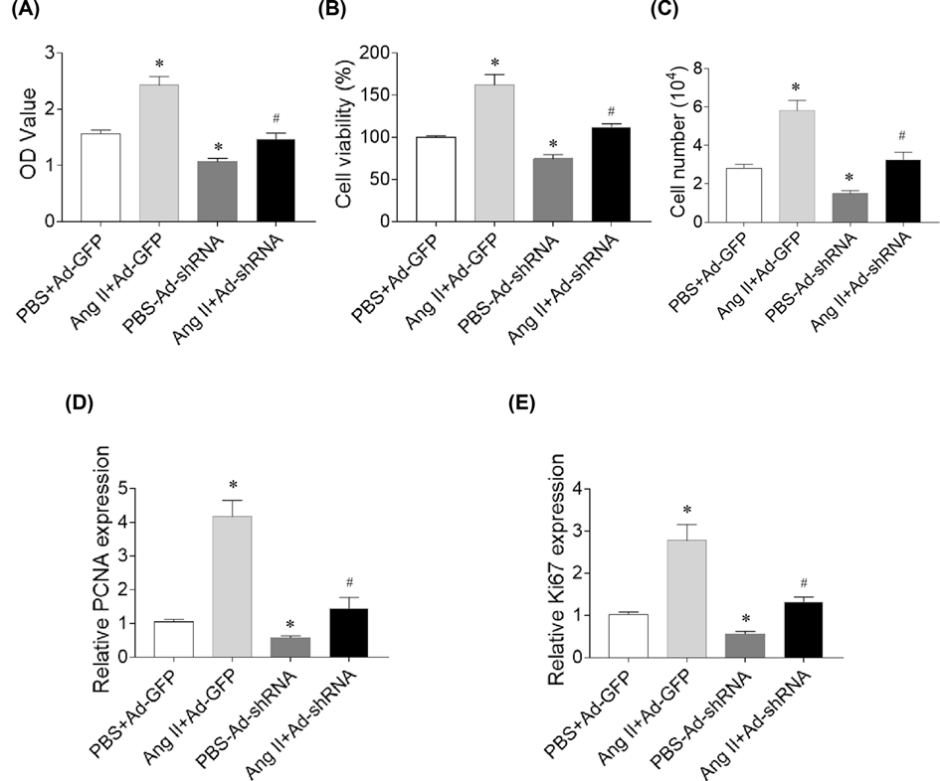
Effects of ATIR down-regulation on the roles of Ang II in SNK-6 cell proliferation and viability (**A**) AT1R down-regulation reversed the effects of Ang II on the increases of proliferation in SNK-6 cell. (**B**) AT1R down-regulation reversed the effects of Ang II on the increases of viability in SNK-6 cell. (**C**) AT1R down-regulation reversed the effects of Ang II on the increases of cell number. (**D**) AT1R down-regulation reversed the effects of Ang II on the increase of PCNA level in SNK-6 cells. (**E**) AT1R down-regulation reversed the effects of Ang II on the increase of Ki67 level in SNK-6 cells. Three independent times experiments were repeated. Results are expressed as mean ± SEM. *P<0.05 versus the PBS+Ad-GFP group; ^#^*P*<0.05 versus the Ang II+Ad-GFP group.

AT1R down-regulation reversed the effects of Ang II on the increases of proliferation (*P*=0.007) and viability (*P*=0.008) of SNK-6 cells. AT1R down-regulation reduced the proliferation (*P*=0.018) and viability (*P*=0.029) of SNK-6 cells (Figure 4A,B). AT1R down-regulation reversed the effects of Ang II on the increases in cell number (*P*=0.002), and AT1R reduced SNK-6 cell number (*P*<0.0001) (Figure 4C). PCNA (*P*=0.028) and ki67 (*P*=0.023) expression levels were reduced by AT1R down-regulation. AT1R down-regulation inhibited the increasing effects on the PCNA (*P*=0.038) and ki67 (*P*=0.040) expression levels in SNK-6 cells (Figure 4).

### Levels of PI3K/Akt

**Figure 5:**
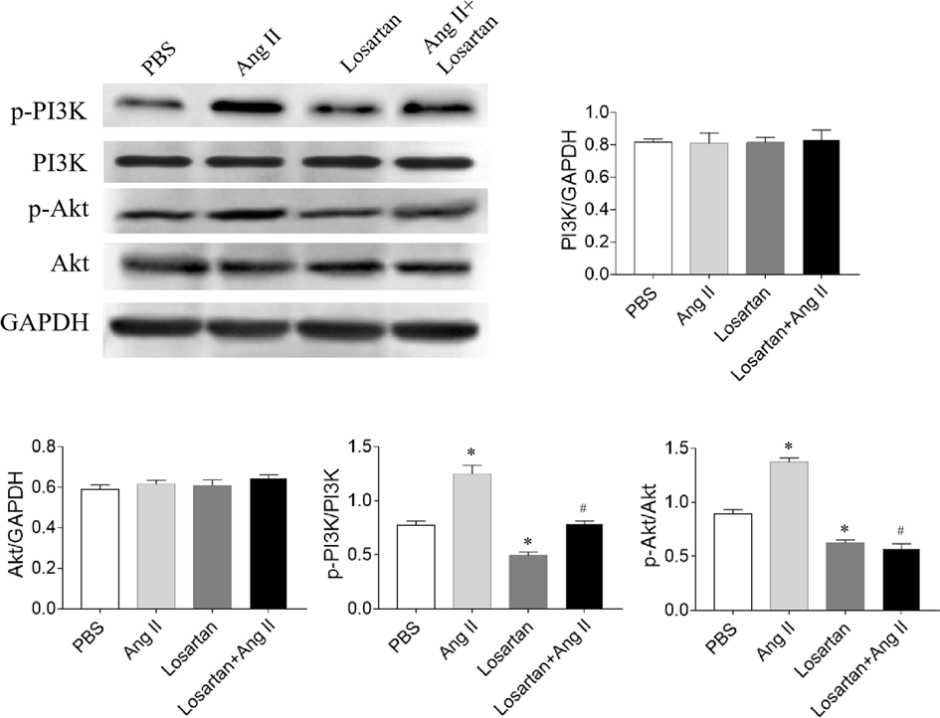
Effects of angiotensin (Ang) II and losartan on phosphatidylinositol 3-kinase/protein kinase B (PI3K/Akt) levels Ang II increased, but losartan reduced the levels of p-PI3K and p-Akt. Three independent times experiments were repeated. Results are expressed as mean ± SEM. *P<0.05 versus the PBS group; ^#^*P*<0.05 versus the Ang II group.

The levels of p-PI3K (*P*=0.026) and p-Akt (*P*=0.036) in SNK-6 cells were increased by Ang II treatment, and decreased by losartan (*P*=0.041 of p-PI3K and *P*=0.045 of p-Akt). Losartan inhibited the increase of p-PI3K (*P*=0.039) and p-Akt (*P*=0.016) after Ang II administration. PI3K and Akt levels showed no significant difference between the four groups (Figure 5).

### Effects of PI3K inhibitor on SNK-6 cell proliferation and viability

**Figure 6:**
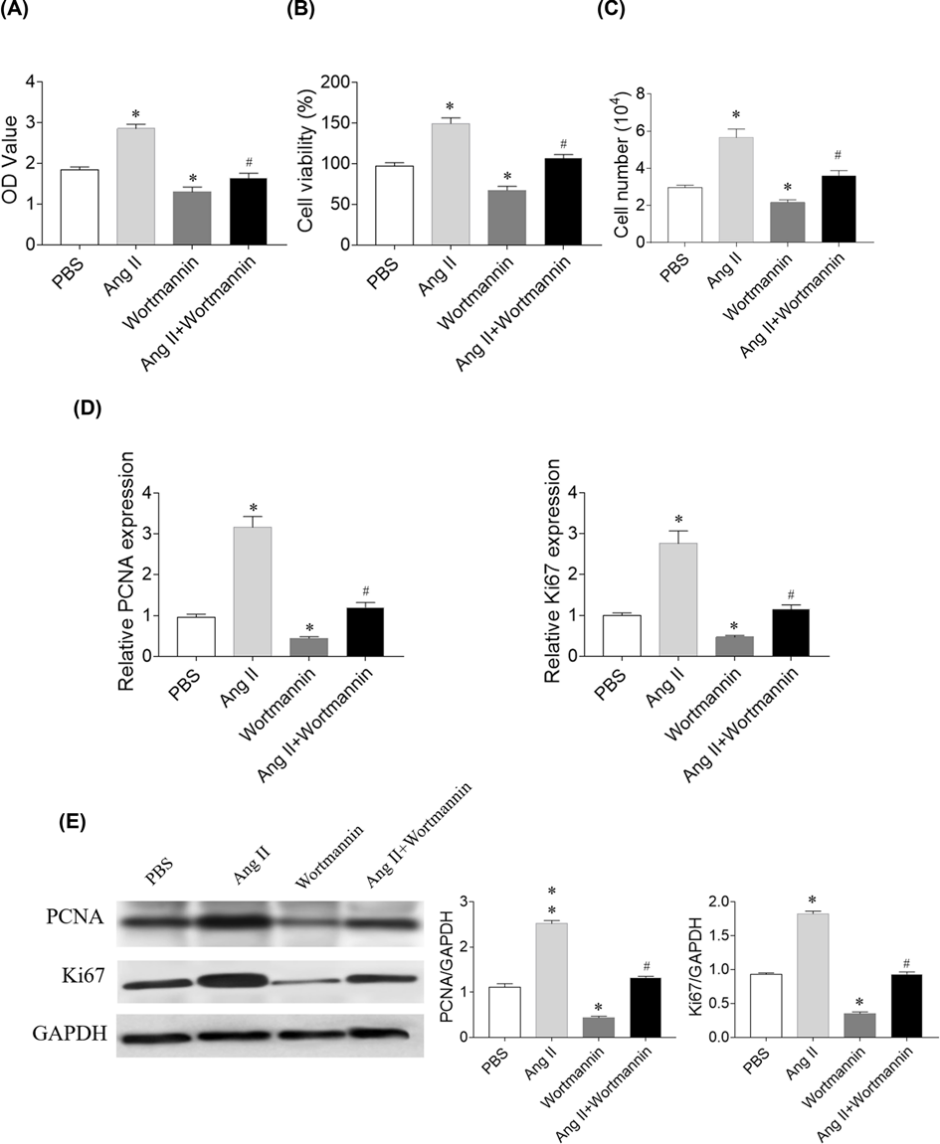
Effects of phosphatidylinositol 3-kinase (PI3K) inhibitors on SNK-6 cell proliferation and viability (**A**) PI3K inhibitor wortmannin reversed the proliferation-boosting effects of angiotensin (Ang) II in SNK-6 cells proliferation with CCK-8 assay. (**B**) Wortmannin reversed the viability-boosting effects of Ang II in SNK-6 cells proliferation with MTT assay. (**C**) Wortmannin reversed the increasing effects of Ang II on the number of SNK-6 cells. (**D**) Wortmannin reversed the effects of Ang II on increasing the mRNA levels of proliferating cell nuclear antigen (PCNA) and Ki67. (**E**) Wortmannin reversed the effects of Ang II on increasing the protein levels of PCNA and Ki67. Three independent times experiments were repeated. Results are expressed as mean ± SEM. *P<0.05 versus the PBS group; ^#^*P*<0.05 versus the Ang II group.

PI3K inhibitor wortmannin (100 nM) reduced the proliferation and viability of SNK-6 cells with CCK-8 (*P*=0.006) and MTT (*P*=0.008) dectection. Wortmannin inhibited the proliferation and viability of SNK-6 cells that had been enhanced by Ang II treatment with CCK-8 (*P*=0.040) and MTT (*P*=0.042) dectection (Figure 6A,B). The increase of SNK-6 cell number induced by Ang II was inhibited by wortmannin (*P*=0.0004), and wortmannin reduced the number of SNK-6 cell (*P*<0.0001) (Figure 6C). Wortmannin reversed the increases of PCNA (*P*=0.030) and Ki67 (*P*=0.033) mRNA levels in SNK-6 cells induced by Ang II (Figure 6D). The increases of PCNA (*P*=0.028) and Ki67 (*P*=0.043) protein levels in SNK-6 cells induced by Ang II were reversed by wortmannin administration (Figure 6E).

### Effects of Akt inhibitor on SNK-6 cell proliferation and viability

**Figure 7:**
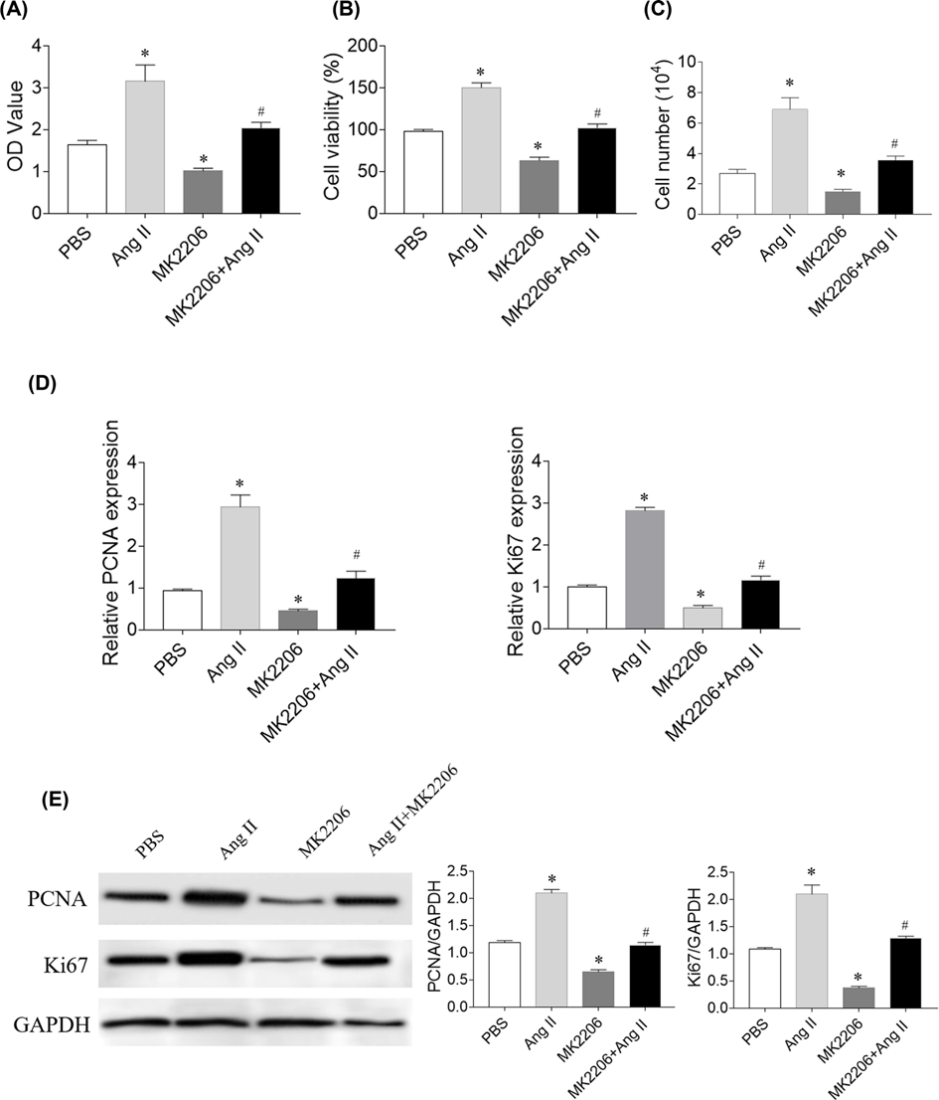
Effects of protein kinase B (Akt) inhibitors on SNK-6 cell proliferation and viability (**A**) Akt inhibitor MK2206 reversed the proliferation-boosting effects of angiotensin (Ang) II in SNK-6 cells proliferation with CCK-8 assay. (**B**) MK2206 reversed the viability-boosting effects of Ang II in SNK-6 cells proliferation with MTT assay. (**C**) MK2206 reversed the increasing effects of Ang II on the number of SNK-6 cells. (**D**) MK2206 reversed the effects of Ang II on increasing the mRNA levels of proliferating cell nuclear antigen (PCNA) and Ki67. (**E**) MK2206 reversed the effects of Ang II on increasing the protein levels of PCNA and Ki67. Three independent times experiments were repeated. Results are expressed as mean ± SEM. *P<0.05 versus the PBS group; ^#^*P*<0.05 versus the Ang II group.

Akt inhibitor MK2206 (5 μM) attenuated the increase in the proliferation and viability of SNK-6 cells induced by Ang II administration with CCK-8 (*P*=0.033) and MTT (*P*=0.029) dectection (Figure 7A,B). MK2206 inhibited the increases of PCNA (*P*=0.043) and Ki67 (*P*=0.032) mRNA levels in SNK-6 cells induced by Ang II (Figure 7C). The increases of PCNA (*P*=0.041) and Ki67 (*P*=0.035) protein levels in SNK-6 cells induced by Ang II were reversed by MK2206 treatment (Figure 7D).

## Discussion

An aggressive Non-Hodgkin’s lymphoma, NK/T-cell lymphoma has a high incidence and a poor outcome in the world [24]. Ang II in RAS is an active peptide regulating tissue remodeling, cell angiogenesis and proliferation [25,26]. Previous studies have shown that RAS is associated with lymphoma [12,27]. Our study found that the expression of Ang II and AT1R increased in lymphoma tissues. Ang II increased the size and weight of lymphoma in nude mice and the proliferation of SNK-6 cells via activating p-PI3K and p-Akt signaling pathway.

AT1 receptor is highly expressed in gastric cancer cell lines and tissues [28]. Ang II and AT1R levels increase in gastric cancer tissues compared with healthy tissues [17]. Ang II facilitates the migration and metastasis of breast cancer cells [21]. In the present study, we found that Ang II level was higher in lymphoma tissue than in control tissue. AT1R expression level also increased significantly in lymphoma tissue as showed in Figure 1. These results indicate that Ang II may be potently involved in the pathogenesis of lymphoma.

Activation of (pro) renin receptor and AT1R triggers the pathogenesis of conjunctival extranodal marginal zone B-cell lymphoma by producing fibroblast growth factor 2 and matrix metallopeptidases [12]. AT1R overexpression is an independent adverse prognosticator for esophageal squamous cell carcinoma (ESCC), and Ang II/AT1R signaling stimulates ESCC growth [29]. In the present study, Ang II treatment increased the size and weight of lymphoma in nude mice, but AT1R antagonist losartan significantly inhibited this trend in nude mice intraperitoneally injected with NK/T-cell lymphoma cells. Furthermore, losartan hampered the increase in tumor volume and weight induced by Ang II treatment as presented in Figure 2. These results indicate that Ang II promotes the growth of lymphoma in mice with immune deficiency.

Ang II can promote the proliferation of human hepatocellular carcinoma HepG2 cells by activating AT1R [30]. Losartan suppresses Ang II-induced cholangiocarcinoma cell proliferation in a dose-dependent manner, and induced cell apoptosis [31]. Ang II treatment enhances the mitogen and anti-CD3-stimulated proliferation of T and NK cells [32]. Here we found that Ang II significantly increased and losartan significantly reduced the proliferation and viability of SNK-6 cells in Figure 3. Losartan and AT1R knockdown inhibited the proliferation and viability of SNK-6 cells strengthened by Ang II treatment as showed in Figures 3 and 4. The levels of PCNA and Ki67 were increased by Ang II, but reduced by losartan administration. These results demonstrate that Ang II enhances and AT1R antagonist losartan significantly reduces lymphoma cell proliferation and viability.

Ang-(1–7) pretreatment inhibits tumor growth via suppressing PI3K/Akt/mTOR pathway [6]. Enforced serine/threonine kinase 33 expression promotes the growth of pancreatic neuroendocrine tumor via activating PI3K/Akt/mTOR pathway [33]. The PI3K/AKT pathway activation is a marker actively implicated in pro-survival cell signaling and cancer progression [34,35]. Also, the PI3K-Akt pathway activation exerts an antiapoptotic effect on neuronal cells in rats with subarachnoid hemorrhage [36]. Moreover, a recent report indicated that the activation of Lyn/PI3K/AKT pathway mitigated cytotoxicity in human lymphocytes under aggressive oxidative stress [37]. In the present study, the levels of p-PI3K and p-Akt in SNK-6 cells increased after Ang II treatment, and this increase was curbed by losartan in Figure 5. PI3K inhibitor wortmannin and Akt inhibitor MK2206 effectively reduced the proliferation and viability of SNK-6 cells as presented in Figures 6 and 7. These results demonstrate that PI3K/Akt can regulate the activity of Ang II in SNK-6 cells proliferation.

In conclusion, Ang II/AT1R may be potently associated with the pathogenesis of lymphoma, and AT1R inhibitors may be used to combat lymphoma. Inhibition AT1R or Ang II synthesis may be a strategy for lymphoma treatment.

## Abbreviations

Akt: protein kinase B
Ang: Angiotensin
AT1R: Ang II type 1 receptor
ECL: chemiluminescence
ELISA: enzyme-linked immunosorbent assay
ESCC: esophageal squamous cell carcinoma
FBS: fetal bovine serum
GAPDH: glyceraldehyde 3-phosphate dehydrogenase
NK/T: Natural Killer/T
NPC: nasopharyngeal carcinoma
PCNA: proliferating cell nuclear antigen
PI3K: phosphatidylinositol 3-kinase
RAS: renin–angiotensin system

## References

[1] Amini R.M. and Enblad G. (2003) Relationship between Hodgkin's and non-Hodgkin's lymphomas. Med. Oncol. 20, 211–220 10.1385/MO:20:3:211 14514970

[2] Schmitz N., Trumper L., Ziepert M. et al. (2010) Treatment and prognosis of mature T-cell and NK-cell lymphoma: an analysis of patients with T-cell lymphoma treated in studies of the German High-Grade Non-Hodgkin Lymphoma Study Group. Blood 116, 3418–3425 10.1182/blood-2010-02-270785 20660290

[3] Pinter M. and Jain R.K. (2017) Targeting the renin-angiotensin system to improve cancer treatment: Implications for immunotherapy. Sci. Transl. Med. 9, eaan5616 10.1126/scitranslmed.aan5616 28978752 PMC5928511

[4] Ahmadian E., Khosroushahi A.Y., Eftekhari A. et al. (2018) Novel angiotensin receptor blocker, azilsartan induces oxidative stress and NFkB-mediated apoptosis in hepatocellular carcinoma cell line HepG2. Biomed. Pharmacother. 99, 939–946 10.1016/j.biopha.2018.01.117 29710494

[5] Shen J., Huang Y.M., Wang M. et al. (2016) Renin-angiotensin system blockade for the risk of cancer and death. J. Renin Angiotensin Aldosterone Syst. 17, 1470320316656679 10.1177/1470320316656679 27402638 PMC5843874

[6] Lin Y.T., Wang H.C., Chuang H.C. et al. (2018) Pre-treatment with angiotensin-(1-7) inhibits tumor growth via autophagy by downregulating PI3K/Akt/mTOR signaling in human nasopharyngeal carcinoma xenografts. J. Mol. Med. (Berl.) 96, 1407–1418 10.1007/s00109-018-1704-z 30374682 PMC7095977

[7] Ye G., Qin Y., Lu X. et al. (2015) The association of renin-angiotensin system genes with the progression of hepatocellular carcinoma. Biochem. Biophys. Res. Commun. 459, 18–23 10.1016/j.bbrc.2015.02.030 25701390

[8] Dezsi C.A. (2014) A review of clinical studies on angiotensin II receptor blockers and risk of cancer. Int. J. Cardiol. 177, 748–753 10.1016/j.ijcard.2014.11.031 25465823

[9] Rasha F., Ramalingam L., Gollahon L. et al. (2019) Mechanisms linking the renin-angiotensin system, obesity, and breast cancer. Endocr. Relat. Cancer 26, R653–R672 10.1530/ERC-19-0314 31525726

[10] Zhao Y., Chen X., Cai L. et al. (2010) Angiotensin II/angiotensin II type I receptor (AT1R) signaling promotes MCF-7 breast cancer cells survival via PI3-kinase/Akt pathway. J. Cell. Physiol. 225, 168–173 10.1002/jcp.22209 20458733

[11] Zhang S. and Wang Y. (2018) Telmisartan inhibits NSCLC A549 cell proliferation and migration by regulating the PI3K/AKT signaling pathway. Oncol. Lett. 15, 5859–5864 29552215 10.3892/ol.2018.8002 PMC5840679

[12] Ishizuka E.T., Kanda A., Kase S. et al. (2014) Involvement of the receptor-associated prorenin system in the pathogenesis of human conjunctival lymphoma. Invest. Ophthalmol. Vis. Sci. 56, 74–80 10.1167/iovs.14-15743 25503453

[13] Wu Y.Y., Wu H.C., Wu J.E. et al. (2019) The dual PI3K/mTOR inhibitor BEZ235 restricts the growth of lung cancer tumors regardless of EGFR status, as a potent accompanist in combined therapeutic regimens. J. Exp. Clin. Cancer Res. 38, 282 10.1186/s13046-019-1282-0 31262325 PMC6604380

[14] Xu J., Pan X. and Hu Z. (2018) MiR-502 mediates esophageal cancer cell TE1 proliferation by promoting AKT phosphorylation. Biochem. Biophys. Res. Commun. 501, 119–123 10.1016/j.bbrc.2018.04.188 29709473

[15] Kim J.H., Kim W.S. and Park C. (2019) Interleukin-6 mediates resistance to PI3K-pathway-targeted therapy in lymphoma. BMC Cancer 19, 936 10.1186/s12885-019-6057-7 31601188 PMC6785854

[16] von Keudell G. and Moskowitz A.J. (2019) The Role of PI3K Inhibition in Lymphoid Malignancies. Current Hematologic Malignancy Rep. 14, 405–413 10.1007/s11899-019-00540-w 31359259

[17] Huang M.M., Guo A.B., Sun J.F. et al. (2014) Angiotensin II promotes the progression of human gastric cancer. Mol. Med. Rep. 9, 1056–1060 10.3892/mmr.2014.1891 24424956

[18] Su Q., Huo C.J., Li H.B. et al. (2017) Renin-angiotensin system acting on reactive oxygen species in paraventricular nucleus induces sympathetic activation via AT1R/PKCgamma/Rac1 pathway in salt-induced hypertension. Sci. Rep. 7, 43107 10.1038/srep43107 28338001 PMC5364504

[19] Tang Q., Wang H., Wang X. et al. (2019) Effect of PI3K/PKB signal pathway inhibitor wortmannin pretreatment on intestinal barrier function in severe acute pancreatitic rats. Adv. Clin. Exp. Med. 28, 1059–1066 10.17219/acem/99910 31414732

[20] Uko N.E., Guner O.F., Matesic D.F. et al. (2020) Akt Pathway Inhibitors. Curr. Top. Med. Chem. 20, 883–900 10.2174/1568026620666200224101808 32091335

[21] Rodrigues-Ferreira S., Abdelkarim M., Dillenburg-Pilla P. et al. (2012) Angiotensin II facilitates breast cancer cell migration and metastasis. PLoS ONE 7, e35667 10.1371/journal.pone.0035667 22536420 PMC3334979

[22] Chang Y., Fu X.R., Cui M. et al. (2019) Activated hippo signal pathway inhibits cell proliferation and promotes apoptosis in NK/T cell lymphoma cells. Cancer Med. 8, 3892–3904 31124291 10.1002/cam4.2174 PMC6639190

[23] Livak K.J. and Schmittgen T.D. (2001) Analysis of relative gene expression data using real-time quantitative PCR and the 2(-Delta Delta C(T)) Method. Methods 25, 402–408 10.1006/meth.2001.1262 11846609

[24] Arber D.A., Orazi A., Hasserjian R. et al. (2016) The 2016 revision to the World Health Organization classification of myeloid neoplasms and acute leukemia. Blood 127, 2391–2405 10.1182/blood-2016-03-643544 27069254

[25] Le Noble F.A., Hekking J.W., Van Straaten H.W. et al. (1991) Angiotensin II stimulates angiogenesis in the chorio-allantoic membrane of the chick embryo. Eur. J. Pharmacol. 195, 305–306 10.1016/0014-2999(91)90552-2 1874278

[26] Muramatsu M., Yamada M., Takai S. et al. (2002) Suppression of basic fibroblast growth factor-induced angiogenesis by a specific chymase inhibitor, BCEAB, through the chymase-angiotensin-dependent pathway in hamster sponge granulomas. Br. J. Pharmacol. 137, 554–560 10.1038/sj.bjp.0704893 12359638 PMC1573517

[27] Koca E., Haznedaroglu I.C., Uner A. et al. (2007) Angiotensin-converting enzyme expression of the lymphoma-associated macrophages in the lymph nodes of Hodgkin's disease. J. Natl. Med. Assoc. 99, 1243–1244, 1246-1247 PMC2574316 18020099

[28] Kinoshita J., Fushida S., Harada S. et al. (2009) Local angiotensin II-generation in human gastric cancer: correlation with tumor progression through the activation of ERK1/2, NF-kappaB and survivin. Int. J. Oncol. 34, 1573–1582 10.3892/ijo_00000287 19424575

[29] Li S.H., Lu H.I., Chang A.Y. et al. (2016) Angiotensin II type I receptor (AT1R) is an independent prognosticator of esophageal squamous cell carcinoma and promotes cells proliferation via mTOR activation. Oncotarget 7, 67150–67165 10.18632/oncotarget.11567 27564102 PMC5341864

[30] Qi R., Lei C.G., Bai Y.X. et al. (2018) Mechanism of angiotensin II (Ang II) on the proliferation of human hepatoma cell line HepG2 cells. Zhonghua Gan Zang Bing Za Zhi 26, 601–606 30317792 10.3760/cma.j.issn.1007-3418.2018.08.008 PMC12769257

[31] Saikawa S., Kaji K., Nishimura N. et al. (2018) Angiotensin receptor blockade attenuates cholangiocarcinoma cell growth by inhibiting the oncogenic activity of Yes-associated protein. Cancer Lett. 434, 120–129 10.1016/j.canlet.2018.07.021 30031758

[32] Jurewicz M., McDermott D.H., Sechler J.M. et al. (2007) Human T and natural killer cells possess a functional renin-angiotensin system: further mechanisms of angiotensin II-induced inflammation. J. Am. Soc. Nephrol. 18, 1093–1102 10.1681/ASN.2006070707 17329576

[33] Zhou B., Xiang J., Zhan C. et al. (2019) STK33 promotes the growth and progression of human pancreatic neuroendocrine tumour via activation of the PI3K/AKT/mTOR pathway. Neuroendocrinology 110, 307–320 31261148 10.1159/000501829

[34] Fruman D.A., Chiu H., Hopkins B.D. et al. (2017) The PI3K Pathway in Human Disease. Cell 170, 605–635 10.1016/j.cell.2017.07.029 28802037 PMC5726441

[35] Vanhaesebroeck B., Guillermet-Guibert J., Graupera M. et al. (2010) The emerging mechanisms of isoform-specific PI3K signalling. Nat. Rev. Mol. Cell Biol. 11, 329–341 10.1038/nrm2882 20379207

[36] Zhuang Z., Zhao X., Wu Y. et al. (2011) The anti-apoptotic effect of PI3K-Akt signaling pathway after subarachnoid hemorrhage in rats. Ann. Clin. Lab. Sci. 41, 364–372 22166507

[37] Ruiz-Medina B.E., Lerma D., Hwang M. et al. (2019) Green barley mitigates cytotoxicity in human lymphocytes undergoing aggressive oxidative stress, via activation of both the Lyn/PI3K/Akt and MAPK/ERK pathways. Sci. Rep. 9, 6005 10.1038/s41598-019-42228-4 30979953 PMC6461650

